# Transcriptome sequencing of purple petal spot region in tree peony reveals differentially expressed anthocyanin structural genes

**DOI:** 10.3389/fpls.2015.00964

**Published:** 2015-11-04

**Authors:** Yanzhao Zhang, Yanwei Cheng, Huiyuan Ya, Shuzhen Xu, Jianming Han

**Affiliations:** Life Science Department, Luoyang Normal UniversityLuoyang, China

**Keywords:** tree peony, spot, transcriptome, flavonoids, differentially expressed genes (DEGs)

## Abstract

The pigmented cells in defined region of a petal constitute the petal spots. Petal spots attract pollinators and are found in many angiosperm families. Several cultivars of tree peony contain a single red or purple spot at the base of petal that makes the flower more attractive for the ornamental market. So far, the understanding of the molecular mechanism of spot formation is inadequate. In this study, we sequenced the transcriptome of the purple spot and the white non-spot of tree peony flower. We assembled and annotated 67,892 unigenes. Comparative analyses of the two transcriptomes showed 1,573 differentially expressed genes, among which 933 were up-regulated, and 640 were down-regulated in the purple spot. Subsequently, we examined four anthocyanin structural genes, including *PsCHS*, *PsF3*′*H*, *PsDFR*, and *PsANS*, which expressed at a significantly higher level in the purple spot than in the white non-spot. We further validated the digital expression data using quantitative real-time PCR. Our result uncovered transcriptome variance between the spot and non-spot of tree peony flower, and revealed that the co-expression of four anthocyanin structural genes was responsible for spot pigment in tree peony. The data will further help to unravel the genetic mechanism of peony flower spot formation.

## Introduction

Petal spot is a group of pigment cells restricted in defined region of petal. It is widely distributed in the eudicots and the monocots, such as in Asteraceae, Brassicaceae, Geraniaceae, Ranunculaceae, Papaveraceae, Malvaceae, Scrophulariaceae, Iridaceae, Liliaceae, and Orchidaceae (Thomas et al., 2009). The spots not only attract pollinators (Eckhart et al., 2006; Goulson et al., 2009), but also impart the ornamental value to the flower. Traditional heredity experiments show that the petal spot is under genetic control (Harland, 1929; Gottlieb and Ford, 1988). However, the molecular mechanism of spot formation is not entirely understood (Davies et al., 2012; Yamagishi, 2013). Tree peony (*Paeonia suffruticosa* Andr.) – a woody shrub of the genus *Paeonia* and family *Paeoniaceae* – is a popular ornamental flower owing to its distinct petal colors. In some peony cultivars, the pigmented cells on the corolla form petal spots and show more intense colors as compared with the background. In China, more than 1000 tree peony cultivars are mainly divided into four geographical groups including Xibei, Zhongyuan, Jiangnan, and Xinan groups (Zhang et al., 2007). Most cultivars of the Xibei group cultivars show a red, brown, or deep purple colored spot at the base of each petal. These cultivars may be the progeny of the wild tree peony species *Paeonia rockii* (Wang et al., 2000). Since, the petal spot enhances the ornamental values of the tree peony flowers; it would be commercially beneficial to understand the mechanism of spot development.

Anthocyanins are the main pigments responsible for spot color, and the spatial and temporal expression of anthocyanin-related genes is related to spot formation. Anthocyanins form a major class of essential pigments in the plant and include derivatives of pelargonidin, cyanidin, and delphinidin, which are responsible for red, purple, and blue coloration, respectively. Chalcone synthase (CHS), chalcone isomerase (CHI), flavonoid 3-hydroxylase (F3H), and flavonoid 3′-hydroxylase (F3′H), dihydroflavonol 4-reductase (DFR), and anthocyanidin synthase (ANS), and UDP flavonoid glucosyl transferase (UFGT) are the well-characterized genes from the anthocyanin biosynthetic pathway (Winkel-Shirley, 2001; Koes et al., 2005). Anthocyanin synthesis is predominantly regulated by regulators from MYB, bHLH, and WD40 families (Ramsay and Glover, 2005; Gonzalez et al., 2008; Schaart et al., 2013). They form regulatory complexes to activate expression of anthocyanin structural genes (Goff et al., 1992; Grotewold et al., 2000). The anthocyanin biosynthetic genes in *Arabidopsis* are regulated by a WD-40 regulator (TTG1), three bHLH regulators (GL3, EGL3, and TT8), and four MYB regulators (AtPAP1, AtPAP2, AtMYB113, and AtMYB114) (Chiu et al., 2010). In some flowers, transcript variance of one or more anthocyanin structural genes was responsible for spot color (Yamagishi, 2013), like in petunia, sequence-specific degradation of CHS RNA lead to star-type red in petal (Koseki et al., 2005). Recently, MYB genes were verified to regulated anthocyanin accumulation in spot, such as *NEGAN* in *Mimulus* (Yuan et al., 2014), *LhMYB12*, and *LhMYB12-Lat* in lilies (Yamagishi et al., 2010, 2014), and *PeMYB11* in Orchidaceae (Hsu et al., 2015). Tree peony has a large, singlular spot at the base of petal, which is stable and has different spot type according to previous researches. Zhang et al. (2007) showed that the abundant accumulation of cyanidin-based glycosides at the basal petal was the primary cause of spot formation in petal. However, molecular mechanism of spot formation is still not clear.

Previous studies of anthocyanin have provided unique insights into the molecular mechanisms of many non-model plant species. In the absence of complete genome sequence (as in tree peony), transcriptomic analysis is an effective method for gaining insights into differentially expressed genes (DEGs). Transcriptome sequencing of the spotted tissues and non-spotted tissues of peony will provide useful insights into the genetics of spot formation. Therefore, we sequenced the transcriptome of petal spot and background of the tree peony flower using Illumina HiSeq2000 platform. We then compared the two transcriptomes and filtered the DEGs. Our main objective was to annotate and analyze the DEGs to identify the candidate genes involved in spot development.

## Materials and Methods

### Tissue Collection and Quantification of Flavonoids

Paeonia suffruticosa Andr. cv. “Jinrong” plants were grown under field conditions in Luoyang Academy of Agriculture and Forestry Sciences (Luoyang, China). As is described by Guo et al. (2004), flower opening stages were divided into five stages, including soft bud stage, pre-opening stage, initial opening stage, half opening stage, and full opening stage. On April 10, 2014, the petals of flower bud (soft bud stage) were sampled, the spot and background in each petal was spliced and pooled, respectively. Each sample (approximate 1 g) was extracted with 40 ml acidic methanol (0.1% hydrochloric acid) at 4°C for 12 h. After centrifugation at 5,000 rpm for 5 min, the supernatant was filtered using a 0.22 μm membrane filter. Anthocyanins were investigated on an Agilent 1100 HPLC equipped with a diode array detector (Agilent Technology) as described by Fan et al. (2012). Total anthocyanin content was measured semi-quantitatively from a simple linear regression using cyanidin-3-O-glucoside (Cy3G) as standard at 520 nm, three biological replicates were performed for anthocyanin content detection.

### cDNA Library Construction and Sequencing

Total RNA was extracted using the modified CTAB method, and was then purified with the RNeasy Plant Mini Kit according to manufacturer’s protocols. The RNA-integrity number (RIN) of each sample was determined using an Agilent 2100 Bioanalyzer. mRNA was enriched from 20 μg total RNA using magnetic beads with Oligo (dT), and was cut into random fragments using fragmentation buffer. First-strand cDNA was synthesized using short fragments as templates and random hexamer primers, followed by synthesis of the second-strand cDNA with dNTPs, RNase H and DNA polymerase I. The double-strand cDNA was purified via magnetic beads, subjected to an end repair process by adding a single nucleotide A (adenine) to the 3′ ends and ligating with sequencing adaptors. The suitable fragments were selected and enriched with PCR amplification as templates. The libraries were validated using Agilent 2100 Bioanalyzer and sequenced using an Illumina HiSeq2000 sequencing platform. The preparation of libraries and sequencing projects were performed at Encode Genomics Bio-Technology Co., LTD. (Suzhou, China). The transcriptome datasets were deposited at the NCBI database under accession numbers SRR1825648 and SRR1825653.

### *De Novo* Assembly and Gene Annotation

Raw reads were filtered by removing adapter sequences and low-quality reads with more than 20% Q < 20 bases. The remaining high-quality reads were assembled de novo using Trinity software (Grabherr et al., 2011). The longest transcript (from alternative splicing transcripts) was selected as the unigene in this study. Functional annotations were performed by homology search against the public databases, including NR and Swiss-Prot database using BLAST with an *E*-value of less than 1*e*-5. Blast2GO (Conesa et al., 2005) was employed to obtain the relevant GO terms based on the NR BLAST results, and WEGO software was used to illustrate the distribution of gene classification. Unigenes were used for query against the COG database to predict and classify functions. Pathway assignments were also carried out based on the Kyoto encyclopedia of genes and genomes (KEGG) database.

### Expression Analysis

The clean reads were aligned to assembled unigenes using Bowtie program (Langmead et al., 2009), the transcription abundance of each unigene was measured by calculating Fragments Per Kilobase of transcript per Million mapped reads (FPKM; Trapnell et al., 2010). DEGs between petal spot and background were identified with the EBSeq package (Leng et al., 2013). Here, we used a threshold for false discovery rate (FDR) significance score <0.01 and absolute value of log2ratio >2 to determine significant differences in gene expression.

### Quantitative RT-PCR Analysis

RNA from the flower spot was extracted and purified as described above. Approximately, 2 μg of total RNA per sample was used to synthesize first-strand cDNA using reverse transcription system (Promega). Quantitative RT-PCR was performed using SYBR Premix Ex Taq (Takara) on ABI 7500 system. Primers used are listed in Supplementary Table S1. The amplification program was performed as 95°C for 2 min, followed by 95°C for 15 s and 60°C for 31 s (40 cycles). Three biological replicates were performed for each gene. The combination of *ubiquitin* and *GAPDH* was used to normalized the qPCR data (Yanjie et al., 2012). The relative expression levels of genes were calculated using the 2^-^ΔΔ^CT^ method (Livak and Schmittgen, 2001). The statistical *p*-value was generated by the paired *t*-test. The statistical significance was defined as *p* < 0.05.

## Results

### Flower Color and Anthocyanin Content

**FIGURE 1:**
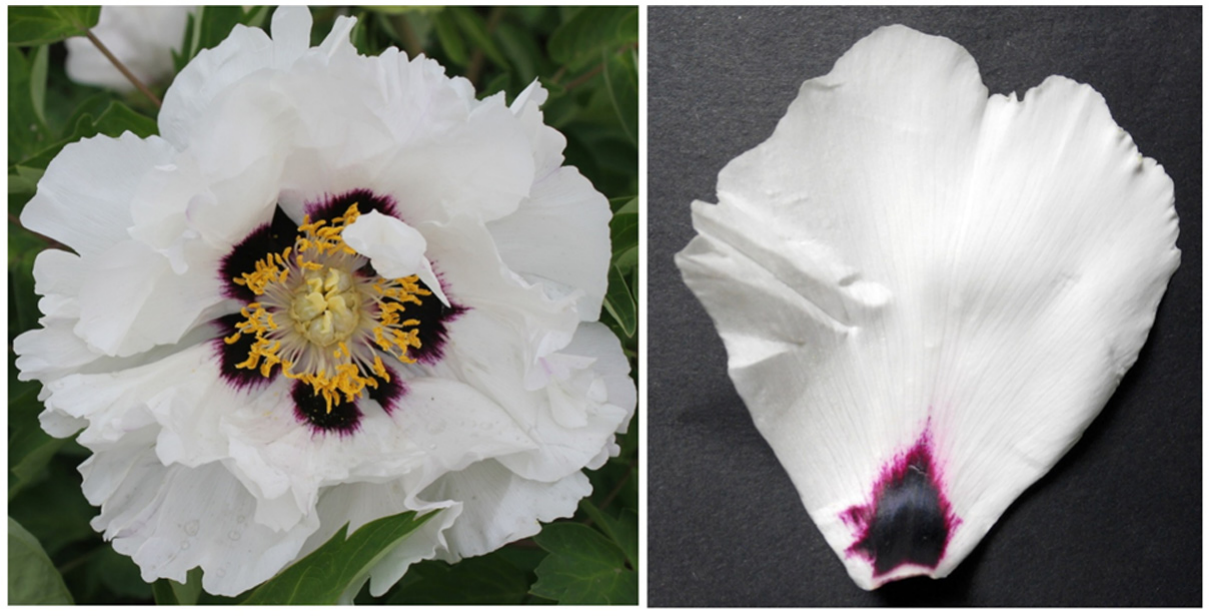
**The flower of tree peony cultivar “Jinrong”**.

The “Jinrong” cultivar of the Xibei group – with a purple spot and white petal – shows significantly demarked spot because of its white background (**Figure 1**). The anthocyanin content of the spot was significantly different from that of the petal. HPLC analysis revealed that highly content of cyanidin-3-*O*-glucoside was accumulated in the spot (1.83 ± 0.07 mg/g), but was barely detectable in the non-spot extraction.

### Transcriptome Sequencing and Assembly

**FIGURE 2:**
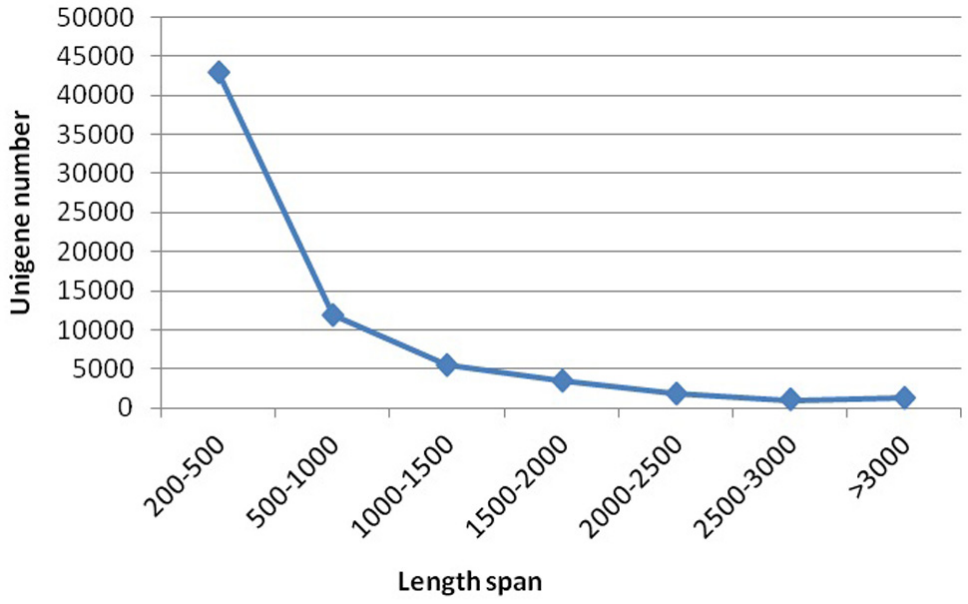
**Length distribution of assembled unigenes.** Please use a single paragraph for each.

Sequencing projects generated 6.95 G and 7.46 G raw data from spot and non-spot libraries, respectively. *De novo* assembly using Trinity Software generated 133,153 transcripts with an N50 of 1,451, and 67,892 unigenes with an N50 of 1,138. A total of 42,860 unigenes (63.13%) were between 200 and 500 nt in length; 11,873 unigenes (17.49%) were between 500 and 1000 nt; and 13,159 unigenes (19.38%) were longer than 1000 nt (**Figure 2**).

### Functional Annotation

All unigenes were annotated by querying against the public databases, including non-redundant protein database (nr), Swiss-Prot, Gene Ontology (GO), KEGG, and the database of Cluster of Orthologous Groups of proteins database (COG). The best hit was selected from the hits with an *E*-value of less than 1*e*-5. Our results showed that 28,039 unigenes were annotated in public databases, accounting for 41.3% of total unigenes. We annotated 27,917 (41.1%), 17,341(25.5%), and 20,800 (30.6%) unigenes to nr, Swiss-Prot, and GO databases, respectively. About 7,980 unigenes (11.8%) and 5,770 (8.5%) unigenes had hits in the COG and KEGG databases, respectively.

### Identification and Annotation of DEGs between Spot and Non-spot Tissues of Tree Peony Petals

**FIGURE 3:**
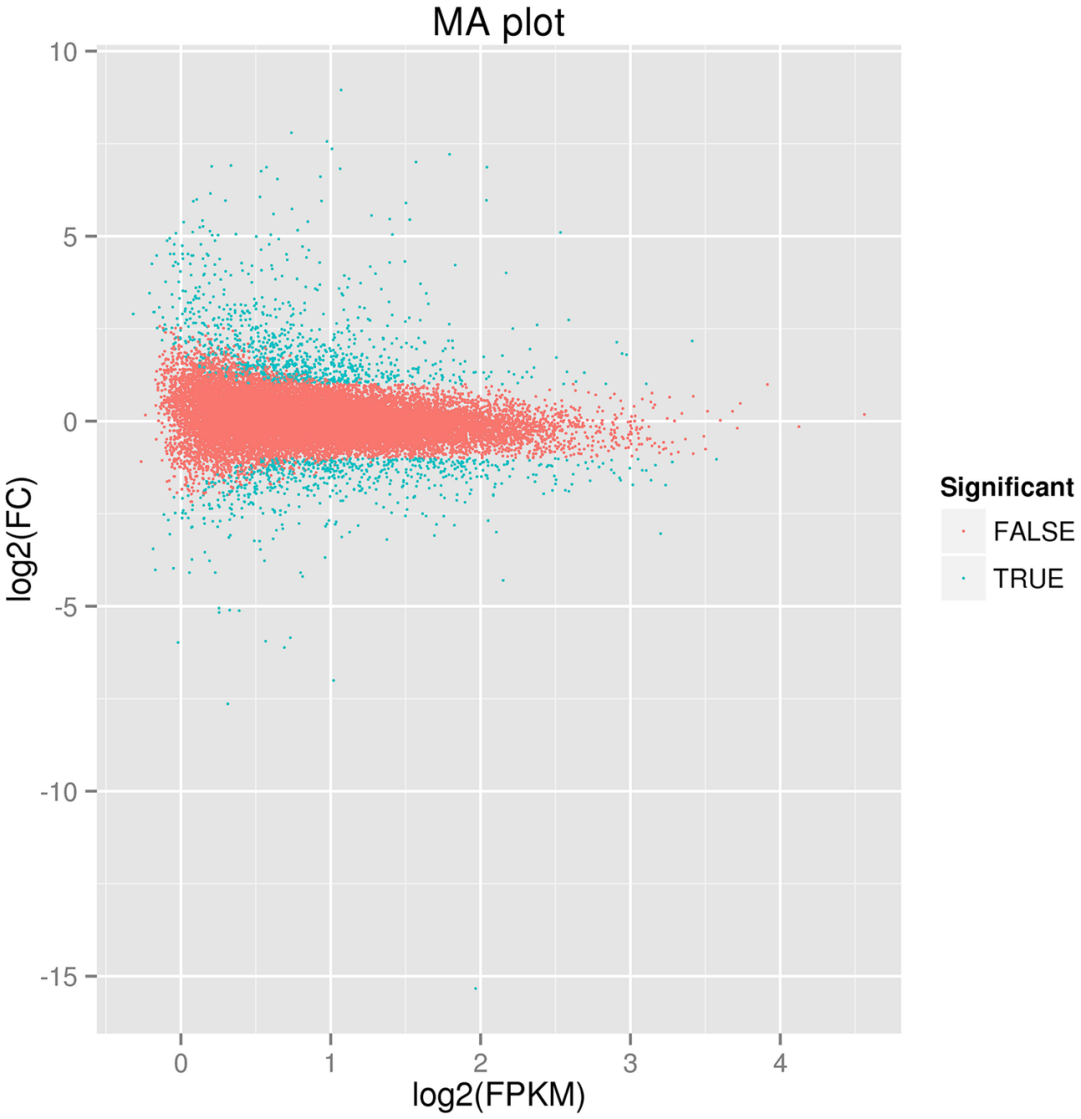
**Figure of differently expressed genes between spot and non-spot.** The parameters “FDR ≤ 0.01” and “log2 ratio ≥ 1” were used as thresholds to determine the different expressed gene. Green dots represent the different expressed genes, and red dots indicate genes that did not change significantly between the two transcriptomes.

**FIGURE 4:**
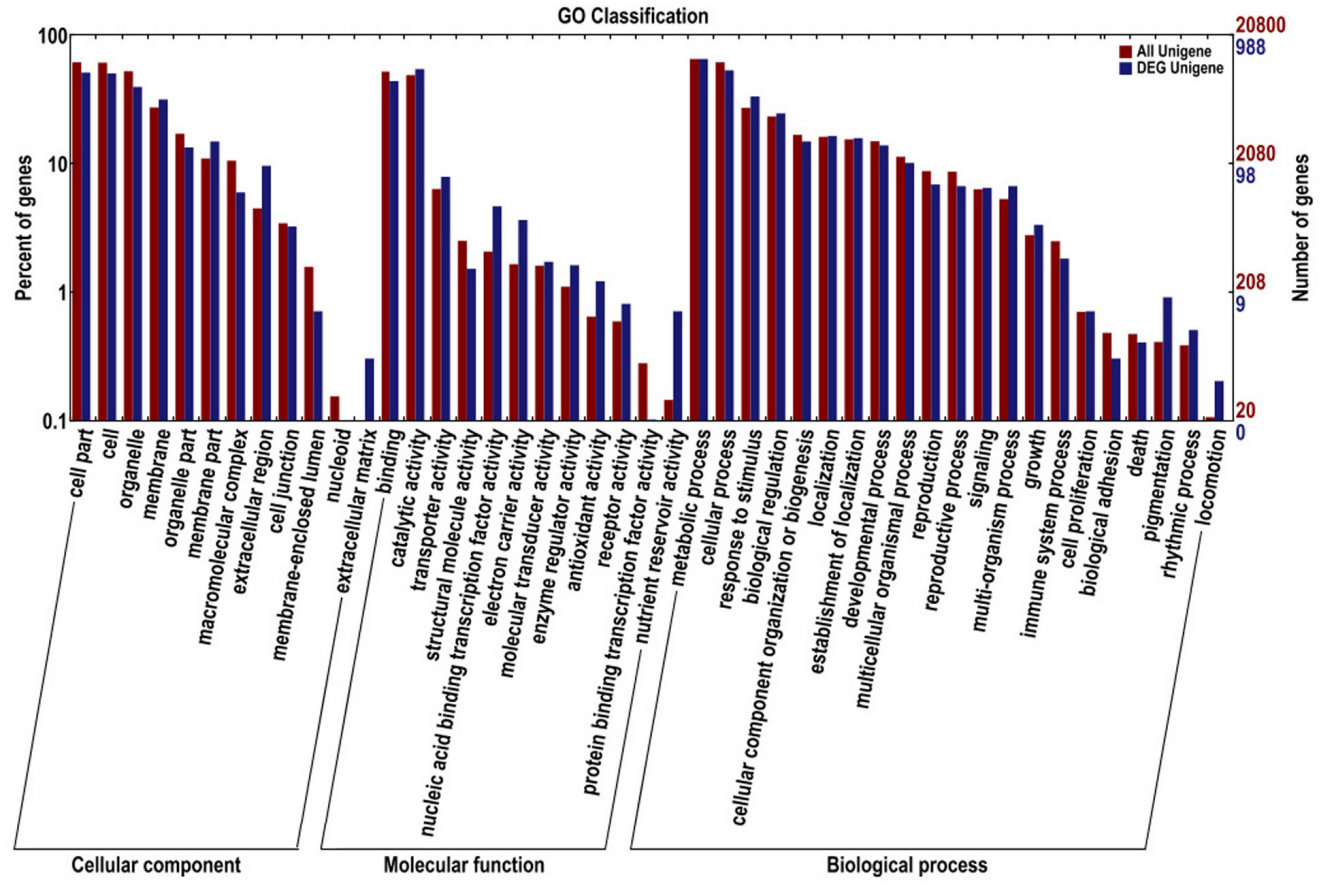
**Gene ontology classifications of assembled unigenes and different expressed genes.** The results are summarized in three main categories: biological process, cellular component, and molecular function.

Comparative transcriptome profiling of the purple spot and non-spot tissues yielded 1,573 DEGs (**Figure 3**). Among these, 933 unigenes were significantly up-regulated while 640 unigenes were down-regulated. We assigned 988 of the 1,573 DEGs to three main GO categories including “molecular functions,” “biological processes,” and “cellular components” (**Figure 4**). Among them, 631 unigenes were grouped in the category “cellular components,” 771 unigenes in “molecular function,” and 792 unigenes in “biological processes.” In the “cellular component” category, “cell part” (505 unigenes), “cell” (498 unigenes), “organelle” (391 unigenes), and “membrane” (312 unigenes) were the most abundant groups. Under the “molecular function” category, the unigenes were most abundant in the “catalytic activity” (538 unigenes) and “binding” (434 unigenes) classes. In the “biological processes” category, the “metabolic process” (645 unigenes) and “cellular process” (526 unigenes) contained more DEGs than others.

### KEGG Classification of DEGs

**FIGURE 5:**
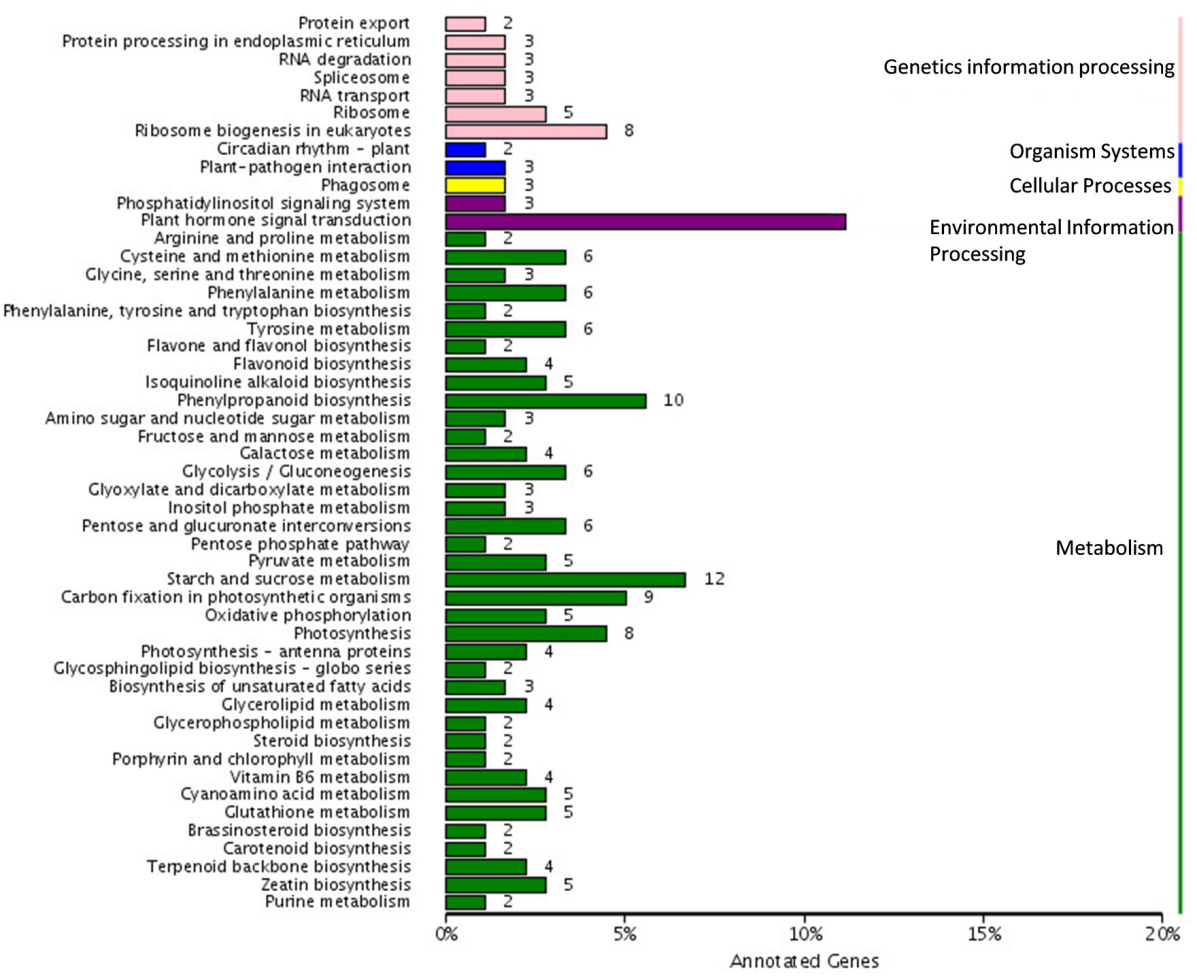
**Pathway assigned of different expressed genes based on Kyoto encyclopedia of genes and genomes (KEGG)**.

We further analyzed the unigenes by searching the KEGG database where out of the 1,573 DEGs, 179 unigenes were assigned to five main categories including 79 pathways (**Figure 5**). Among the five main categories, “metabolism” was the largest category containing 122 unigenes, followed by “genetic information processing” (29 unigenes), “environmental information processing” (23 unigenes), “organismal systems” (5 unigenes), and “cellular processes” (5 unigenes). Since, the spot pigment was correlated with anthocyanin accumulation; we were interested in the anthocyanin biosynthesis pathway. We identified three pathways including “phenylpropanoid biosynthesis” (10 DEGs, ko00940), “flavonoid biosynthesis” (4 DEGs, ko00941), and “flavone and flavonol biosynthesis” (2 DEGs, ko00942) in the metabolism category, which were related to color development. Moreover, there were seven glucide metabolic pathways, including “starch and sucrose metabolism (12 DEGs, ko00500),” “glycosaminoglycan degradation (1 DEG, ko00531),” “amino sugar and nucleotide sugar metabolism (3 DEGs, ko00520),” “pentose and glucuronate interconversions (6 DEGs, ko00040),” “glycolysis/gluconeogenesis (6 DEGs, ko00010),” “fructose and mannose metabolism (2 DEGs, ko00051),” and “galactose metabolism (4 DEGs ko00052).” All of these metabolic pathways may relate to the synthesis of substrates for anthocyanin biosynthesis.

### Transcription Analysis of Anthocyanin Biosynthesis Genes

By annotation in public database, seven genes were predicted to participate in flavonoid pathway (**Table 1**). Among them, c38856.graph_c0 (KT758291), c50492.graph_c0 (KT758292), and c56659.graph_c0 (KT758293) encode F3′H, DFR, and ANS, respectively. They were the same genes with that reported previously in tree peony (Zhang et al., 2014; Zhao et al., 2015). c29075.graph_c0 (KT758290) has 85.3% amino acid similarity with CHS (KJ466964) in tree peony. Phylogenetic analysis revealed that c56800.graph_c0 was more closely related to *FLS* gene in *Arabidopsis*, c61446.graph_c0 and c58959.graph_c0 was not in the clad with UDP-glucose flavonoid 3-*O*-glucosyltransferases that were verified to catalyzes the transfer of the glucosyl moiety from UDP-glucose to the 3-hydroxyl group of anthocyanidins (Supplementary Figures S1 and S2). Therefore, four anthocyanin structural genes, *PsCHS*, *PsF3*′*H*, *PsDFR*, and *PsANS* were identified from DGEs.

**Table 1:** Putative anthocyanin structural genes identified from differentially expressed genes (DEGs).

Unigene	Annotation	FPKM non-spot	FPKM-spot	Log2Ratio (spot/non-spot)
c29075.graph_c0	Chalcone synthase (CHS)	3.0	216.6	6.0
c56800.graph_c0	Flavanone 3-hydroxylase	832.9	407.3	-1.2
c38856.graph_c0	Flavanone 3′-hydroxylase	1.3	66.3	5.4
c50492.graph_c0	Dihydroflavonol-4-reductase	43.7	285.9	2.5
c56659.graph_c0	Leucoanthocyanidin dioxygenase	15.2	282.2	4.0
c61446.graph_c0	UDP-glucose flavonoid 3-*O*-glucosyltransferase	2.1	6.5	1.4
c58959.graph_c0	UDP-glucose flavonoid 3-*O*-glucosyltransferase	17.0	5.0	-2.0

**FIGURE 6:**
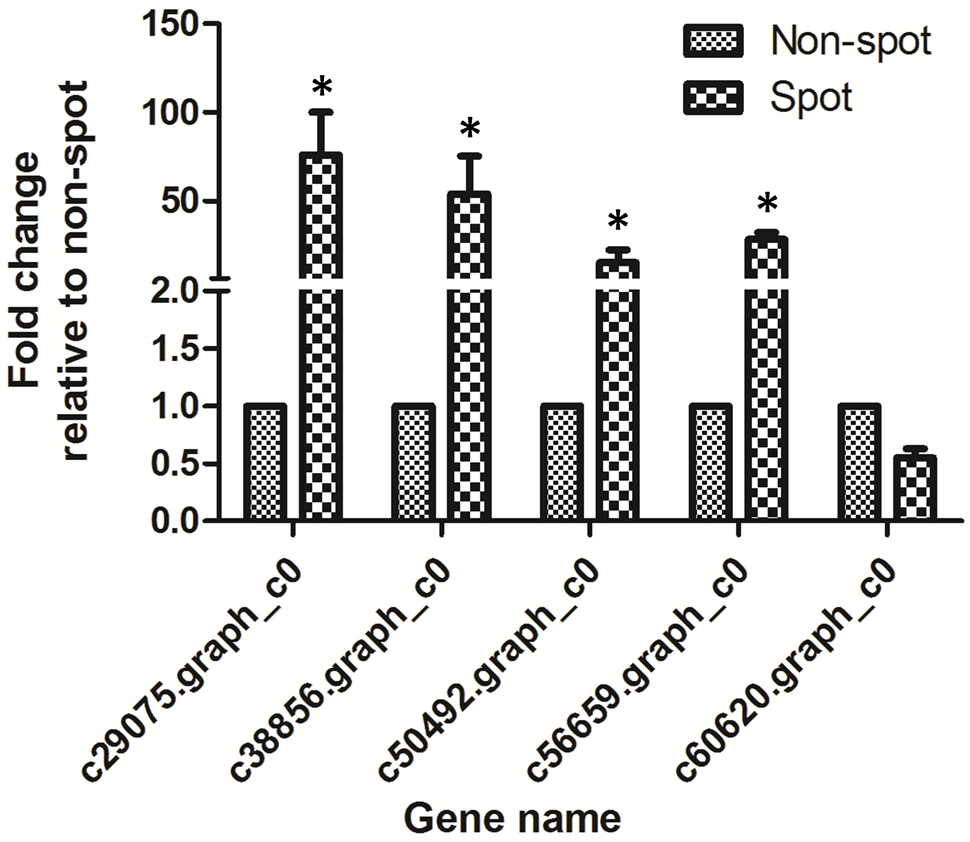
**qRT-PCR analysis of anthocyanin structural genes expression in petal spot and non-spot of “Jinrong”.** *Ubiquitin* was used as an internal control. Each gene has three biological replicates. The statistical *p*-value was generated by the paired *t*-test. Asterisk indicated statistical significance (^∗^*p* < 0.05).

The normalized expression levels of *PsCHS*, *PsF3*′*H*, *PsDFR*, and *PsANS* in spot vs. non-spot were 73.4-, 51.8-, 6.5-, and 18.6-fold higher, respectively. In order to validate the expression profiling by Illumina sequencing, we further analyzed the anthocyanin structural genes using qRT-PCR (**Figure 6**; Supplementary Figure S3). Our results showed that the four genes including *PsCHS*, *PsF3*′*H*, *PsDFR*, and *PsANS* showed significantly higher expression in spot than in non-spot. Thus, our qRT-PCR results were consistent with those using Illumina sequencing method.

In the anthocyanin biosynthesis pathway, structural genes are controlled by transcription factors from MYB, bHLH, and WD40 families (Davies et al., 2012; Robinson and Roeder, 2015). We identified 7 MYBs, 12 bHLHs, and 1 WD40 among the DEGs (**Table 2**). Among these four MYBs, nine bHLHs, and one WD40 were up-regulated in spot. Phylogenetic analysis revealed that c60620.graph_c0 (KT758294) was closely related to VvMYBA1 and VvMYBA2, both of which regulated anthocyanin biosynthesis in grape (Supplementary Figure S4). No bHLH and WD40 genes involved in anthocyanin regulation were found (Allan et al., 2008). However, c60620.graph_c0 was down regulated in spot both by transcriptome and qPCR analysis (**Table 2**, **Figure 6**).

**Table 2:** Differentially expressed genes in transcription factor families of MYB, bHLH, and WD40.

Unigene	Annotation	FPKM non-spot	FPKM spot	Log2Ratio (spot/non-spot)
c47873.graph_c0	Myb domain protein 17 isoform 1	0.6	2.9	2.0
c23531.graph_c0	Myb-like transcription factor family protein	2.4	6.3	1.2
c40196.graph_c0	Myb domain protein 7, putative	0	2.2	4.5
c56086.graph_c0	myb-related protein 306 isoform 1	3.3	9.2	1.2
c51134.graph_c0	Transcription factor MYB113-like	10.8	3.4	–1.9
c37122.graph_c0	Myb-related protein 305	1.8	0.3	–2.5
c60620.graph_c0	Transcription factor MYB114	9.7	4.9	–1.2
c45268.graph_c0	Transcription factor bHLH145	8.5	21.4	1.1
c38652.graph_c0	Transcription factor bHLH155	15.2	6.0	–1.5
c54560.graph_c0	Transcription factor bHLH147	43.4	106.9	1
c44717.graph_c0	Transcription factor bHLH51	0.2	2.7	3.4
c46824.graph_c0	Transcription factor bHLH123	0.5	2.7	2.2
c46115.graph_c0	Transcription factor bHLH135	105.7	249.6	1.0
c45804.graph_c0	Transcription factor bHLH61	1.1	12.3	3.2
c57113.graph_c0	Transcription factor bHLH122	1.5	4.7	1.5
c57019.graph_c0	Transcription factor bHLH79	4.4	10.6	1.1
c39077.graph_c0	Transcription factor bHLH120	18.6	3.2	–2.7
c45948.graph_c0	Transcription factor bHLH117	0	1.8	4.8
c61342.graph_c0	WD-repeat protein, putative	7.9	18.4	1.0

## Discussion

In this study, we found four key anthocyanin structural genes including *PsCHS*, *PsF3*′*H*, *PsDFR*, and *PsANS* were expressed at a significantly higher level in the red spot than in the white non-spot. HPLC analysis showed that the anthocyanin content was consistent with color variance between the spot and non-spot. Anthocyanins are tightly linked to color variation in the flower. In petal spot, the color cue is usually determined by anthocyanin accumulation. Cyanidin-based glycosides accumulate abundantly at the basal petal in tree peony and result in spot formation (Zhang et al., 2007). Therefore co-expression of the four structural genes identified in this study is responsible for pigmentation in petal spot of “Jinrong”.

Tree peony lacks a reference genome, and thus restricts the molecular understanding of spot formation. Next generation sequencing technologies allow a large scale retrieval of DEGs in non-model plants lacking a reference genome. In this study, we sequenced and comparatively analyzed the transcriptomes of the spot and non-spot using Illumina. We assembled 67,892 unigenes and annotated1573 DEGs. Pathway analysis studies annotated DEGs to 78 metabolic pathways. The “flavonoid biosynthesis” (ko00941) and “flavone and flavonol biosynthesis” (ko00944) pathways relevant to pigment development were annotated. Moreover, we also annotated seven glucide metabolic pathways that may synthesize substrate for anthocyanin. Anthocyanins and their co-pigments are responsible for color formation in flowers. The pathways involved in the production of both anthocyanins and their co-pigments were described in transcriptomic analysis of plants like *Brassica juncea* (Liu et al., 2013), peach flower (Chen et al., 2014), grape hyacinth (Lou et al., 2014), herbaceous peony (Zhao et al., 2014), and Saﬄower (Lulin et al., 2012). Our results indicated that flavonoid and anthocyanin biosynthetic pathways were conserved in tree peony.

In the anthocyanin biosynthetic pathway, CHS is responsible for the first committed step. It condenses one molecule of 4-coumaroyl-CoA with three molecules of malonyl-CoA to produce the tetrahydroxy-chalcone, which is the precursor for the biosynthesis of flavonoids and anthocyanins. CHI converts the tetrahydroxychalcone to naringenin. F3H, F3′H, and F3′5′H catalyzes the formation of dihydroflavonols from naringenin. Dihydroflavonols are converted by DFR, ANS, and flavonoid 3-glucosyl transferase (UF3GT) to yield pelargonidin, cyanidin, and delphinidin pigments (Winkel-Shirley, 2001; Lepiniec et al., 2006). Plant color variation is attributed to variation in transcripts of structural genes, such as in *Clarkia gracilis* (Martins et al., 2013), *Litchi chinensis* Sonn (Wei et al., 2011), *Magnolia sprengeri* Pamp (Shi et al., 2014), and torenia plants (Nishihara et al., 2014). Our transcriptome profiling study showed that *CHS*, *F3*′*H*, *DFR*, and *ANS* showed higher expression at a significantly higher level on the spot than in the non-spot. These results were further corroborated by qRT-PCR analysis. Our result suggests that the color variance mechanism is conserved in plants. High expression levels of structural genes ensure sufficient anthocyanin accumulation to make the spot purple, while the opposite effect is prevalent in the non-spot.

Flowers color is usually determined by activation of anthocyanin pathway. MYB-bHLH-WD40 were the key regulatory genes responsible for flower color patterns. Spots are common pigmentation pattern in flower, and some arbitrary and variable spots were caused by transposable elements insertion in anthocyanin structural or regulatory genes (Iida et al., 2004; Nishijima et al., 2013). Recently, MYB genes are likely involved in controlling the spot formation, such as *NEGAN* in monkey flowers (Yuan et al., 2014) and *PeMYB11* in Orchidaceae (Hsu et al., 2015). Tree peony has another type of spot, which is a large, singular spot at the base of petal. In *C. gracilis* and pansy, a spot similar with tree peony was present in petal, researches revealed that the precise spatio-temporal expression of *DFR2*, *F3*′*H1* in spot, and *DFR1* and *F3*′*5*′*H1* genes in background lead to spot formation in *C. gracilis* (Martins et al., 2013), while transcription of *VwF3*′*5*′*H*, *VwDFR*, and *VwANS* was significantly increased in cyanic blotches of pansy, but which is the critical regulatory genes was still unclear (Li et al., 2014). As is reviewed by Davies et al. (2012) and Yamagishi (2013), spots appearing at the same position, may or may not be associated with specific epidermal cell types, are shaped by the most complex regulatory mechanisms and the molecular mechanisms are not fully understood. We suggested that the co-expression of anthocyanin structural genes was responsible for anthocyanin accumulation in spot, but transcription variance of structural genes between spot and non-spot may not be directly caused by transcription variance of regulated genes from MYB, bHLH, and WD40 families. We did not found a MYB genen from DEGs responsible for the up-regulation of anthocyanin pathway genes. However, our data provide more clues to reveal the mechanism of activation of anthocyanin pathway in spot.

## Conflict of Interest Statement

The authors declare that the research was conducted in the absence of any commercial or financial relationships that could be construed as a potential conflict of interest.
