# Supplementary material for: Transcriptome sequencing of purple petal spot region in tree peony reveals differentially expressed anthocyanin structural genes
Source: Front Plant Sci. 2015 Nov 4;6:964. doi: 10.3389/fpls.2015.00964 (PMC4631938; doi:10.3389/fpls.2015.00964)
Supplement: Supplementary file 1 [file Data_Sheet_2.DOC]

***Supplementary Material***

**Transcriptome sequencing of purple petal spot region in tree peony reveals differentially expressed anthocyanin structural genes**

**Yanzhao Zhang^1†^, Yanwei Cheng^1†^*, Huiyuan Ya^1†^ , Shuzhen Xu^1^, Jianming Han^1^**

^1^ Life Science Department, Luoyang Normal University, Luoyang 471022, China

*** Correspondence:** Yanwei Cheng, Life Science Department, Luoyang Normal University, Luoyang 471022, China.

[ywei_cheng@163.com](mailto:ywei_cheng@163.com)

†These authors contributed equally to this work

**Supplementary Table**

**Table S1 Primers used for qRT-PCR**.

| Gene ID | Forward primer | Reverse primer |
| --- | --- | --- |
| c29075.graph_c0 | GTCGGTGGCTCAGACAATAG | AAATAAGTCCAGGCACATCC |
| c38856.graph_c0 | AACTTGTTCACGGCAGGGAC | GAAGGGGAGATTTGGGAGGT |
| c50492.graph_c0 | AGCCAACAATAAATGGAATG | GGATCTTGGACACGAAATAC |
| c56659.graph_c0 | TAGAACAAGAAGTCGGTGGC | GTGGAGGATGAAAGTGAGGG |
| c58959.graph_c0 | CCTTCTTATTTGTTCTACGCTT | GTAACTCGGGACGATTGACT |
| c61446.graph_c0 | ATTTGGGGTCCCTACTTACG | AGAGGGGAGTACCTTTGTCG |
| c56800. graph_c0 | CCATCGACAGTGCAAGCAAAG | GTACACTTCGTTTGCCGCTCT |

**Supplementary Figures**


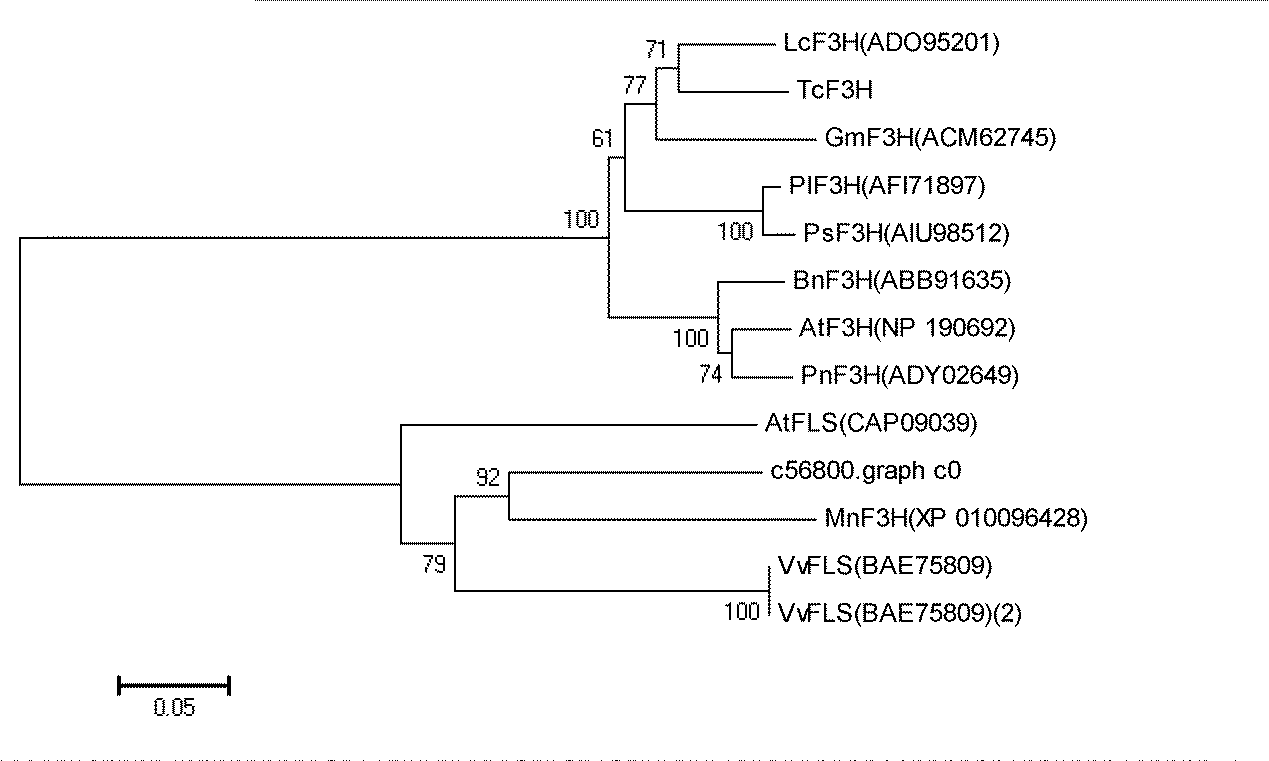


**Fig S1. Phylogenetic analysis of F3H in tree peony and others plant species.** Full-length protein sequences were aligned using Clustal W, phylogenetic analysis was conducted with MEGA 5.0 using Neighbor-joining method and 1000 bootstrap replicates.


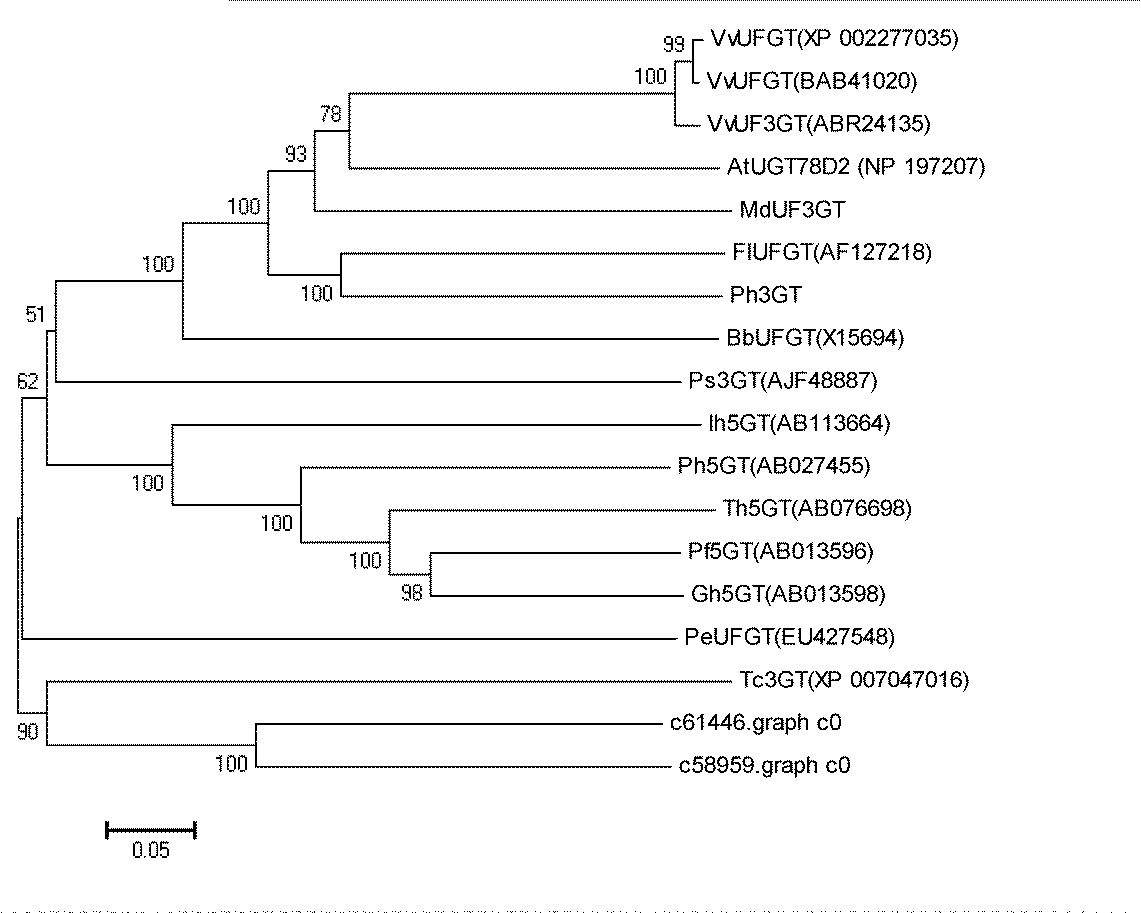


**Fig S2.** **Phylogenetic analysis of UF3GT in tree peony and others plant species.** Full-length protein sequences were aligned using Clustal W, phylogenetic analysis was conducted with MEGA 5.0 using Neighbor-joining method and 1000 bootstrap replicates.

**
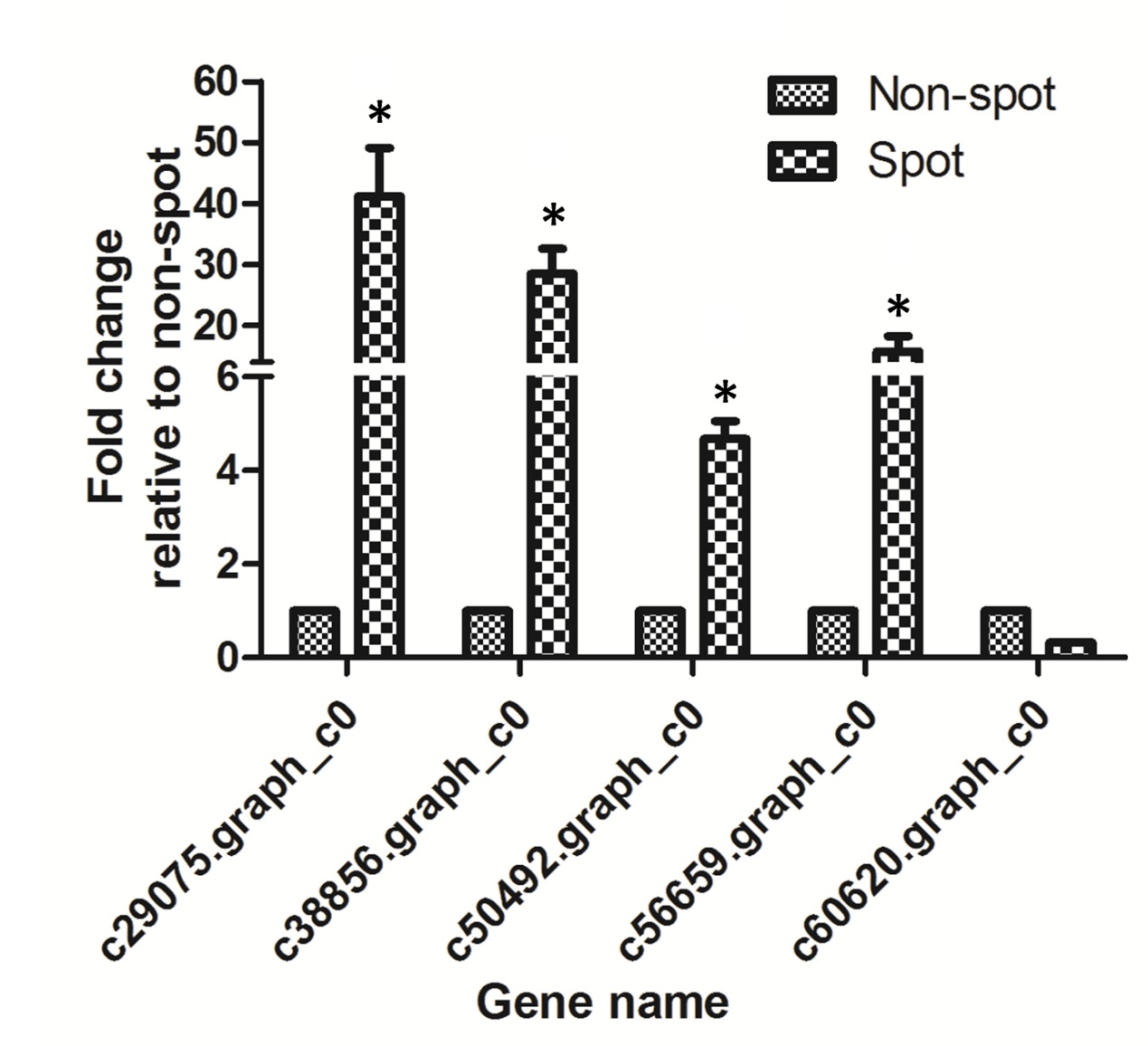
**

Figure S3．qRT-PCR analysis of anthocyanin structural genes expression in petal spot and non-spot of "Jinrong". *GADPH* was used as an internal control. Each gene has three biological replicates. The statistical P value was generated by the paired t-test. Asterisk indicated statistical significance (p <0.05).

**
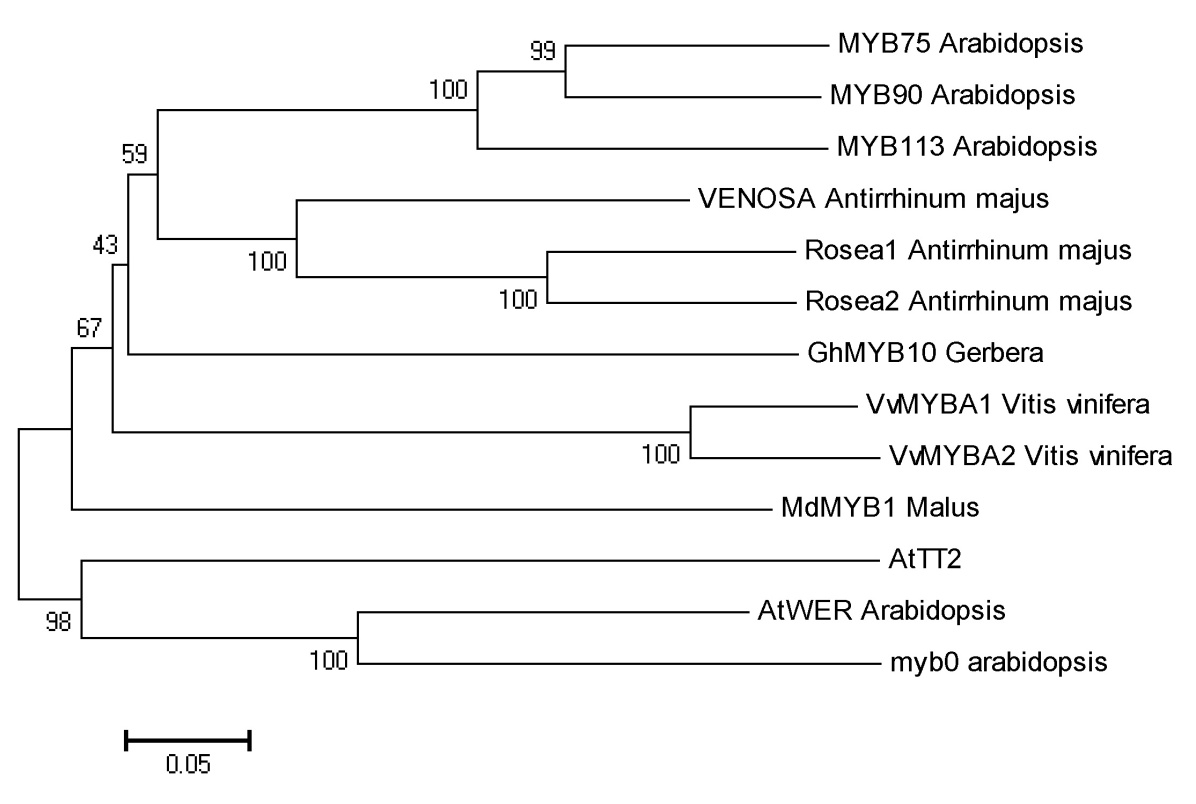
**

**Fig S4. Phylogenetic analysis of anthocyanin regulated MYB gene in tree peony and others plant species.** Full-length protein sequences were aligned using Clustal W, phylogenetic analysis was conducted with MEGA 5.0 using Neighbor-joining method and 1000 bootstrap replicates.
